# Supplementary material for: Engineering topological states in atom-based semiconductor quantum dots
Source: Nature. 2022 Jun 22;606(7915):694–9. doi: 10.1038/s41586-022-04706-0 (PMC9217742; doi:10.1038/s41586-022-04706-0)
Supplement: Supplementary file 1 — This Supplementary Information file includes Supplementary Tables 1–6, Figs. 1–5 and References. [file 41586_2022_4706_MOESM1_ESM.pdf]

nature portfolio

https://doi.org/10.1038/s41586-022-04706-0

Supplementary information

Engineering topological states in atom-based semiconductor quantum dots

In the format provided by the authors and unedited

Supplementary Materials: Engineering topological states in atom-based semiconductor quantum dots

M. Kiczynski,[1, 2] S. K. Gorman,[1, 2] H. Geng,[1, 2] M. B. Donnelly,[1, 2]
Y. Chung,[1, 2] Y. He,[1, *] J. G. Keizer,[1, 2] and M. Y. Simmons[1, 2]
[1]*Centre of Excellence for Quantum Computation and Communication Technology, School of Physics, UNSW Sydney, Kensington, New South Wales 2052, Australia*
[2]*Silicon Quantum Computing Pty Ltd., Level 2, Newton Building, UNSW Sydney, Kensington, New South Wales 2052, Australia*
(Dated: May 9, 2022)

I. PRECISE ENGINEERING OF QUANTUM DOT SIZE, TUNNEL COUPLINGS AND CONTROL GATES

An overview of the main parameters of the devices are presented in Table S1. The quantum dot sizes and inter-dot distances are directly measured from the STM images. The nanoscale size of the quantum dots allow us to achieve large on-site energies ($>$ 20 meV); and the small separation of $\leq$ 10 nm allow us to achieve large tunnel couplings $t \approx 1 - 10$ meV. With the nanoscale accuracy of STM lithography we can engineer the trivial and topological phases so that we can change $\langle v/w \rangle$ from 0.265 to 2.08.

A. Quantum dot size

The quantum dot sizes in the devices are engineered to be $\sim$ 25 nm^2 hosting $\sim$ 25 P donors per site [1]. These sizes were chosen as this on-site energy is robust against small variations in donor numbers within the quantum dots as shown in Fig. S1. Here we plot the on-site energies as a function of the dot size using electrostatic modelling and assuming a 1.5 ± 0.5 nm decay of the donor wavefunction outside the lithographic size of the dot (as shown by the shaded grey region). Generally, for small quantum dot sizes with only a few P donors (for example 1P, 2P and 3P) we can see from Fig. S1 that a change in a single P number results in a large variation in U_i [2]. This variation is not as important for large quantum dots with $>$ 15 donors. For larger quantum dots, however, the capacitive coupling between the quantum dots become too large and the dots start to behave as a single large quantum dot or wire [3]. From the experimental zero-bias conductance peaks (see Fig. 3e of the main text) we can determine the average on-site energy of the quantum dot arrays using lever arms determined from the electrostatic modelling [4]. The measured $\langle U \rangle = 32.9 \pm 3.8$ meV for device I is above the expected value based on the electrostatic modelling, whereas $\langle U \rangle = 22.0 \pm 3.2$ meV for device II is below. These values are consistent with charging energies of previously measured quantum dots [5], with the differences between dots attributed to small variations in the gate structure (and hence lever arms, α, device I: $\langle \alpha \rangle = 0.2550 \pm 0.0221$ and device II: $\langle \alpha \rangle = 0.2574 \pm 0.0197$), and quantum dot sizes (device I: 27.7 ± 2.9 nm^2 and device II: 23.0 ± 2.8 nm^2). These effects can all contribute to different charging energies calculated based on the constant interaction model [3]. Note, on the same device, using the same STM tip, the variation in dot size was less than 13%.

B. Inter-dot tunnel couplings

The tunnel coupling follows an exponential dependence on the inter-dot separation [8]. Not only do we have to engineer alternating tunnel couplings for the SSH simulation, the inter-dot separation must also be close enough that there is sufficient transport current through the device to perform bias spectroscopy. However, we cannot make the tunnel and capacitive couplings between the quantum dots too large as they will not behave independently [3]. Watson *et al.* [1] have previously demonstrated electron transport through a triple donor quantum dot, with lithographic sizes of approximately 4 x 4 nm^2, with $\sim$ 15 P donors. Here the separations between the quantum dots were $\sim$ 9.5 nm and $\sim$ 10.5 nm and the source/drain leads were $\sim$ 10 nm from the array. Using such an arrangement it was possible to achieve sufficient transport currents through this triple quantum dot system with $>$ 1 nA at a bias of 2 mV. Utilising this knowledge we restricted quantum dot separations between $6 \leq d_i \leq 12$ nm to ensure the quantum dots are spaced far enough apart such that they behave independently but close enough that sufficient current can be measured through the device. These distances allowed us to engineer a large enough ratio between the alternating tunnel couplings to investigate the trivial and topological phases of the SSH model. We note that this is challenging in larger devices due to the exponential dependence of the tunnel coupling with dot separation.

* Current address: Shenzhen Institute for Quantum Science and Engineering, Southern University of Science and Technology, Shenzhen, 518055, China

TABLE S1. **Device parameters for the Trivial (Device I) and Topological (Device II) phases.** The average quantum dot size (nm^2) and inter-dot distances, $\langle d_v \rangle$ and $\langle d_w \rangle$ (nm), directly measured form the STM images, the on-site energy, $\langle U \rangle$ (meV), measured from the experimental zero-bias conductance peaks, the lever arms, $\langle \alpha \rangle$, calculated from the electrostatic modelling and tunnel couplings, $\langle v \rangle$ and $\langle w \rangle$ (meV), determined from fitting the experimental zero-bias conductance peaks.

	⟨ Dot size ⟩	$\langle d_v \rangle$	$\langle d_w \rangle$	$\langle U \rangle$	$\langle \alpha \rangle$	$\langle v \rangle$	$\langle w \rangle$	$\langle v/w \rangle$
Trivial	27.7 ± 2.9	7.7 ± 0.1	10.1 ± 0.2	32.9 ± 3.8	0.2550 ± 0.0221	4.89 ± 0.40	2.35 ± 0.23	2.08
Topological	23.0 ± 2.8	9.6 ± 0.4	7.8 ± 0.6	22.0 ± 3.2	0.2574 ± 0.0197	1.39 ± 0.35	5.25 ± 2.02	0.265

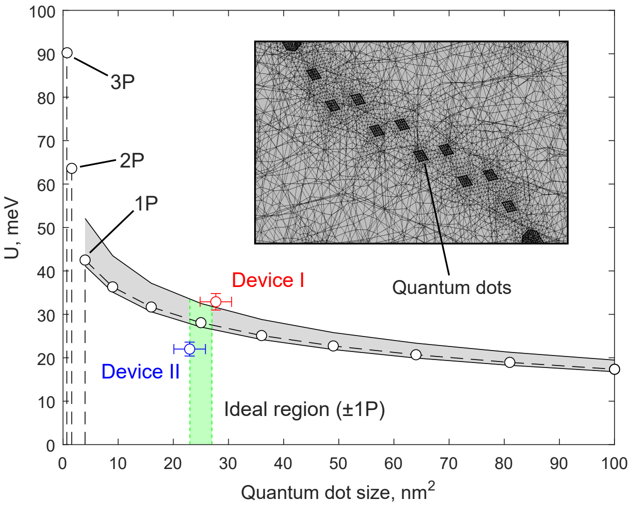

FIG. S1. **Optimisation of the on-site energy U based on the donor quantum size.** Theoretically calculated on-site energies, U, based on COMSOL electrostatic modelling as a function of the quantum dot size (black circles). The black line is a $1/d$ fit to the theoretical data, with the shaded grey region corresponding to a ± 0.5 nm variation in the 'seam'. The quantum dot size chosen for the experiment is shown by the centre green dashed region with the outer green lines corresponding to ± 1 donor. The vertical dashed black lines correspond to a 3P, 2P, and single donor (in increasing quantum dot size). The variation of U for a single donor changes significantly from $U_{1P} \approx 45$ meV [6] to $U_{2P} \approx 65$ meV [2, 7] for a 2P quantum dot. The variation in U is much smaller for the 25 nm^2 quantum dot size used in the main text with an expected change of only ±1 meV. The blue (topological phase device) and red (trivial phase device) circles are the measured $\langle U \rangle$ determined using the calculated lever arms from the electrostatic modelling. The inset shows the electrostatic mesh used to model the quantum arrays indicating the location and area of the quantum dots.

C. Electrostatic gate density

To tune the energy levels of the different quantum dots in the array we require control gates capacitively coupled to the quantum dots that can both tune each quantum dot's energy levels independently but also globally. In order to tune the energy levels of each quantum dot independently would typically require at least 10 control gates. However, for realising the SSH model this is not necessary as it is only important to bring the energy levels into resonance at zero source/drain bias. With precision donor devices the low density all-epitaxial gates can be patterned with nanometre accuracy and can be separated far enough away to allow reasonable operating gate ranges (> 1 V) before breakdown leakage occurs. The addition of more than the 6 patterned gates does not achieve better independent control of the energy levels of the individual quantum dots, since the gates become equally capacitively coupled to the same quantum dots. Instead, it is more important to be able to engineer sufficient differential lever-arms between the various gates to the quantum dots.

To maximise both the absolute and differential lever-arms, the layout of a 10 quantum dot array with the required gates was considered. Figure S2a, b and c, show a schematic of a segment of the array with the dots arranged in a linear, right-angled and tilted arrangement respectively. The linear arrangement, whilst preventing parallel tunnelling through the array, results in the lowest differential lever-arms with the ability to achieve independent control of neighbouring quantum dots greatly reduced [4]. To improve this differential coupling the quantum dots can be arranged in a tilted array arrangement as shown in Fig. S2c, with maximal differential control occurring when the dots are arranged at right angles to each other (Fig. S2b). However, in this right-angled arrangement the next-nearest neighbour distance between the quantum dots is also reduced resulting in parallel tunnelling through the quantum dots, which is not allowed in the SSH model, which requires sequential tunnelling through the array. As such, in order to prevent parallel tunnelling through the array, while still maximising the differential lever-arms, the dots were arranged in a tilted array with a 120° angle. In this arrangement next-nearest neighbour tunnelling is exponentially suppressed with estimated $t_{i,i+2}/t_{i,i+1} \approx 0.01$, ensuring electron transport occurs sequentially through the array.

Figure S2d shows a schematic of the device layout with the position of the control gates relative to the 10 quantum dot array. Importantly the layout of the gates are rotationally symmetric on either side of the array such that combined lever arm from all gates to the quantum dots is approximately equal allowing for the array to be globally controlled. For the gate layout; the number, position and sizes of the gates were considered. With the restriction on the absolute length of the array set by the tunnel couplings it is possible to engineer 6 control gates into the device. To maximise the absolute lever-arms the gates were positioned as close as possible to the

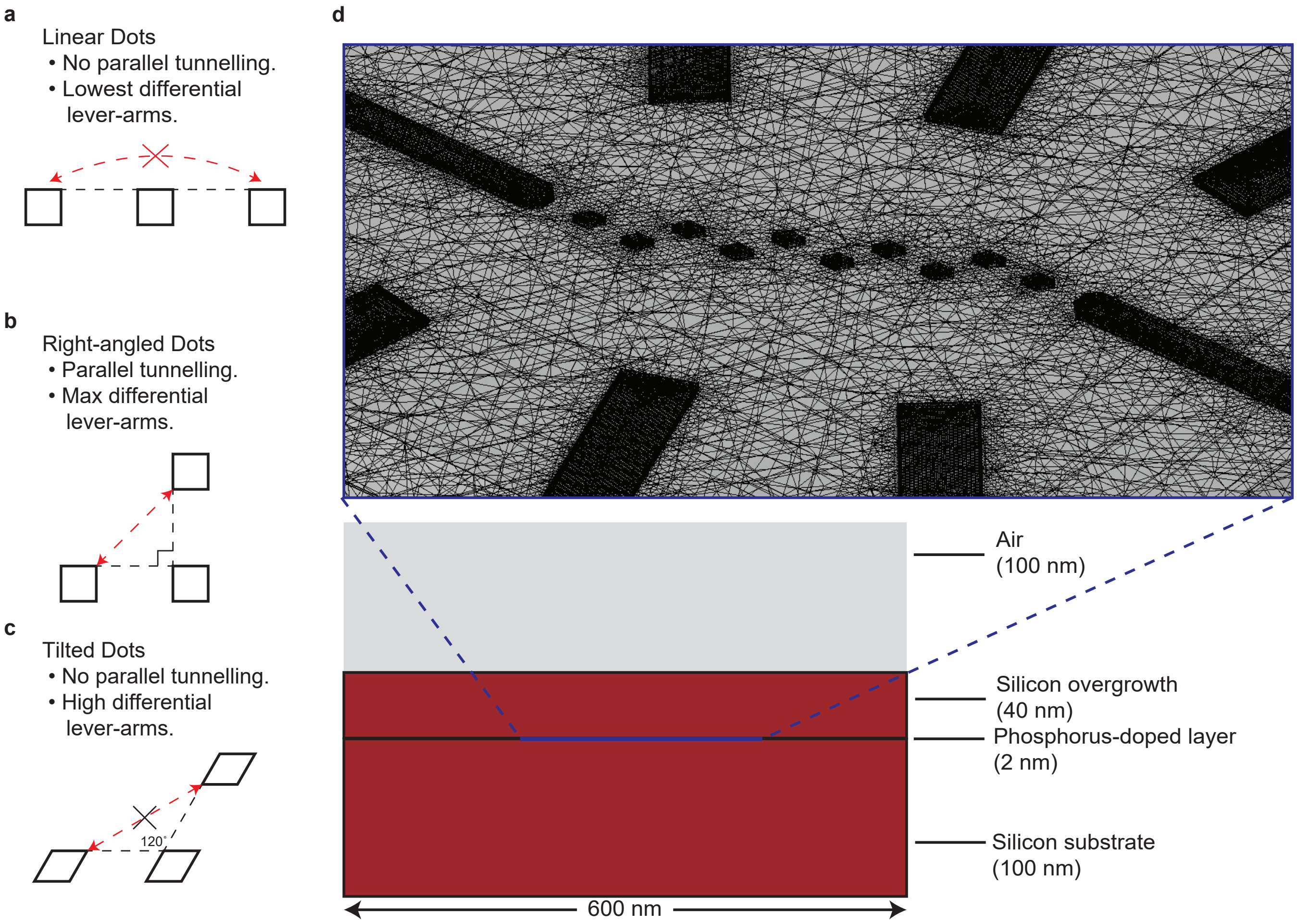

FIG. S2. **Engineering global and differential electrostatic control of nanoscale quantum dot arrays.** **a**, A linear arrangement of quantum dots results in the lowest differential lever-arms and greatly reduces the ability to independently tune of the quantum dot energy levels. **b**, A right-angled arrangement of the quantum dots has the greatest differential tunability of the quantum dots, however, allows parallel tunnelling through the array. **c**, A tilted arrangement of the quantum dots as used our devices has highly independent tunability whilst suppressing parallel tunnelling. **d**, Overview of the capacitance mesh of the device design used, which has a linear array of 10 quantum dots, in a tilted arrangement, as shown in c, tunnel coupled to source and drain leads, with 6 control gates, $\sim$20 nm wide, placed $\sim$ 55 nm from the array, capacitively coupled to the quantum dots. A cross-sectional view of the device layer structure is shown below with the 40 nm silicon overgrowth and 2 nm thick phosphorus doped layer. Here we add a 100 nm silicon substrate below and a 100nm air gap on top for the capacitance simulations.

TABLE S2. **Trivial phase, Device I lever-arms.** Lever-arms of each gate, labelled G1 to G6, to each quantum dot, labelled D1 to D10, along with the total lever-arm of all gates acting on each dot.

	D1	D2	D3	D4	D5	D6	D7	D8	D9	D10
G1	0.0785	0.0758	0.0397	0.0383	0.0256	0.0242	0.0168	0.0157	0.0130	0.0112
G2	0.0540	0.0856	0.0522	0.0781	0.0473	0.0619	0.0350	0.0399	0.0255	0.0238
G3	0.0195	0.0294	0.0262	0.0430	0.0375	0.0653	0.0474	0.0776	0.0519	0.0645
G4	0.0106	0.0116	0.0138	0.0146	0.0203	0.0220	0.0318	0.0329	0.0592	0.0591
G5	0.0261	0.0264	0.0418	0.0360	0.0636	0.0507	0.0805	0.0556	0.0878	0.0563
G6	0.0661	0.0494	0.0727	0.0443	0.0609	0.0372	0.0407	0.0260	0.0284	0.0191
Total	0.2547	0.2781	0.2465	0.2544	0.2552	0.2612	0.2522	0.2477	0.2658	0.2340

TABLE S3. **Topological phase, Device II lever-arms.** Lever-arms of each gate, labelled G1 to G6, to each quantum dot, labelled D1 to D10, along with the total lever-arm of all gates acting on each dot.

	D1	D2	D3	D4	D5	D6	D7	D8	D9	D10
G1	0.0712	0.0741	0.0417	0.0424	0.0275	0.0271	0.0196	0.0184	0.0144	0.0122
G2	0.0468	0.0727	0.0493	0.0748	0.0481	0.0649	0.0402	0.0471	0.0292	0.0270
G3	0.0180	0.0263	0.0244	0.0385	0.0346	0.0587	0.0472	0.0775	0.0533	0.0652
G4	0.0116	0.0130	0.0161	0.0169	0.0231	0.0259	0.0398	0.0412	0.0737	0.0733
G5	0.0282	0.0287	0.0447	0.0376	0.0604	0.0498	0.0799	0.0548	0.0818	0.0520
G6	0.0652	0.0499	0.0699	0.0430	0.0537	0.0353	0.0405	0.0261	0.0279	0.0185
Total	0.2409	0.2646	0.2461	0.2532	0.2474	0.2615	0.2671	0.2652	0.2803	0.2481

TABLE S4. **Trivial phase, Device I, Coulomb interaction terms, in meV.** Diagonal terms are the on-site, U_i, Coulomb terms and the off-diagonal terms are the inter-site, $V_{i,j}$, Coulomb terms, with $V_{i,j} = V_{j,i}$, between the dots labelled D1 to D10.

	D1	D2	D3	D4	D5	D6	D7	D8	D9	D10
D1	31.6341	4.0979	2.0602	1.2905	0.8514	0.6052	0.4325	0.3241	0.2306	0.1540
D2	-	32.3460	3.7095	2.2958	1.3315	0.9359	0.6406	0.4791	0.3330	0.2220
D3	-	-	32.4018	4.6171	2.4129	1.4646	0.9704	0.6925	0.4771	0.3110
D4	-	-	-	33.5702	3.9327	2.3162	1.3523	0.9533	0.6271	0.4077
D5	-	-	-	-	33.9613	4.3813	2.2971	1.4266	0.9109	0.5676
D6	-	-	-	-	-	31.9503	3.9703	2.3344	1.2947	0.7935
D7	-	-	-	-	-	-	34.0740	4.5279	2.2352	1.2070
D8	-	-	-	-	-	-	-	33.3486	3.7508	1.9399
D9	-	-	-	-	-	-	-	-	33.4493	3.9548
D10	-	-	-	-	-	-	-	-	-	32.1580

TABLE S5. **Topological phase, Device II, Coulomb interaction terms, in meV.** Diagonal terms are the on-site, U_i, Coulomb terms and the off-diagonal terms are the inter-site, $V_{i,j}$, Coulomb terms, with $V_{i,j} = V_{j,i}$, between the dots labelled D1 to D10.

	D1	D2	D3	D4	D5	D6	D7	D8	D9	D10
D1	20.8626	2.3461	1.3279	0.7643	0.5460	0.3759	0.2799	0.2011	0.1485	0.0944
D2	-	22.6319	2.9451	1.5354	0.9697	0.6439	0.4577	0.3260	0.2351	0.1488
D3	-	-	22.7265	2.6728	1.6006	0.9327	0.6505	0.4422	0.3179	0.1972
D4	-	-	-	22.0545	3.1461	1.5964	0.9693	0.6395	0.4373	0.2691
D5	-	-	-	-	21.8433	2.7527	1.5313	0.8911	0.5974	0.3539
D6	-	-	-	-	-	22.8299	2.8517	1.4889	0.8721	0.5024
D7	-	-	-	-	-	-	23.0762	2.5861	1.4215	0.7251
D8	-	-	-	-	-	-	-	21.6729	2.6286	1.2272
D9	-	-	-	-	-	-	-	-	21.1172	2.3171
D10	-	-	-	-	-	-	-	-	-	21.1956

array ($\sim$ 55 nm), and made as wide as possible ($\sim$ 20 nm), while still keeping the distances far apart to allow reasonable operating gate ranges without leakage. The gates were spaced around the array to maximise the differential lever-arm of the gates, while also having the combined lever-arm of all the gates to each quantum dot relatively consistent such that the potential of all dots can be shifted together. With this arrangement; gate 1 is most strongly coupled to quantum dots 1, 2, and 3, with gate 4 most strongly coupled to quantum dots 10, 9, and 8, gate 2 to quantum dots 4, 2, and 6, gate 5 to quantum dots 7, 9 and 5, and gate 3 to quantum dots 8, 10 and 6, and gate 6 to quantum dots 3, 1 and 5.

To estimate the lever-arms, α, and the on-site, U_i, and inter-site, $V_{i,j}$, Coulomb interactions of the device, electrostatic modelling was performed using COMSOL Multiphysics [4, 9], an electrostatic finite element solver was used to calculate the Maxwell capacitance matrix for the device. We assume a phosphorus layer thickness of 2 nm and a 1.5 nm 'seam' around each element of the device representing the spread of the donor wavefunction outside of the patterned lithographic structures. From the constant interaction model [3] and the capacitance matrix the lever-arms, α, on-site, U_i, and inter-site, $V_{i,j}$, Coulomb interaction terms can be calculated. The Coulomb interaction terms are then scaled with respect to the experimentally measured on-site energies such that $\langle U_{measured}\rangle = \langle U_{comsol}\rangle$. The lever-arms for each dot in device I, in the trivial phase, and device II, in the topological phase, are listed in Table S2 and S3 respectively. The Coulomb interaction terms, in meV, for device I and II are listed in Tables S4 and S5 respectively, where the diagonal terms are the on-site terms, U_i, and the off diagonal terms are the inter-site, $V_{i,j}$, Coulomb interaction terms, with $V_{i,j} = V_{j,i}$. These values were calculated using the exact distances measured with the STM during fabrication and fit to the measured conductance peaks (see Methods).

II. HIGH-BIAS ($V_{sd} \neq 0$) REGIME

For sufficiently small source-drain bias, $V_{sd} < t_{i,i+1}, V_{i,i+1}, U_i$ only a single electron can tunnel through the quantum dots since multi-electron tunnelling processes require multiple states to be within the bias window of the source and drain leads. This is the regime investigated in the main text. In this section, we show large bias stability diagrams to investigate the system when $V_{sd} \neq 0$. In particular, we look at the stability diagram of both devices over large voltage ranges $\sim$ 1 V corresponding to the addition of $>$ 50 electrons (see conduction peaks in Fig. S3c and f). Examination of the full stability diagrams elucidates the differences between the two phases of the SSH model.

As stated in the main text the quantum dots can be described by the extended Hubbard model with the Hamiltonian,

$$H_U = \sum_{i=1}^{N} \epsilon_i n_i + \sum_{i}^{N} U_i n_i (n_i - 1) + \sum_{i}^{N-1} t_{i,i+1}(c_i^\dagger c_{i+1} + h.c.) + \sum_{i,j}^{N} V_{i,j} n_i n_j, \quad (1)$$

where ϵ_i are the energy levels of the ith dot of the array, n_1 the dot occupation operator, $t_{i,i+1}$ are the tunnel coupling between nearest neighbour ith and $ith+1$ dots, U_i is the on-site Coulomb interaction term, $V_{i,j}$ is the inter-site Coulomb interaction terms between the ith and jth sites, and $h.c.$ indicates Hermitian conjugate.

In Fig. 3a of the main text we showed the theoretical calculation of the conductance through the quantum dot array as a function of the tunnel coupling ratio (v/w) between the quantum dots. For this calculation we considered the regime where there can only be at most 1 electron on each of the quantum dots (large U-limit). This large U regime means we can neglect the upper Hubbard band in the calculations. The inter-site Coulomb interactions, $V_{i,j}$ cause breaking of particle-hole symmetry and gives rise to $N!$ possible conduction pathways. For our 10 quantum dot devices, $N = 10$ we therefore have a total of 3628800 different *undecuple* points in gate space where electrons can move through the array. Note that depending on the strength of $V_{i,j}$ many of these pathways may be close to degenerate, for example $V_{1,10} \approx 0$ since the quantum dots are separated by $>$ 50 nm and would therefore not be experimentally observable due to small energy differences between these two conduction paths. To assess the validity of the large U approximation we measured the stability diagram of each device over a large voltage range shown in Fig. S3.

In the experiment we do not know the absolute electron numbers on each of the quantum dots; however, we know the inner electrons on each quantum dot do not participate in the electron transport. Instead, these inner electrons contribute an energy offset to the total energy of the Hamiltonian [10]. The resulting Coulomb ladders for large gate voltages (V $\sim$ 1000 mV) are shown in Fig. S3 for both devices. The stability diagrams shown in Fig. S3a, b, d, e are measured by first aligning the quantum dots at a particular set of gate voltages (noted as Align. in Fig. S3a and d) and then sweeping the combined gate voltages. Therefore, the further away from the fully aligned voltage position the more detuned the quantum dots will be and there will be contributions from other conductance pathways through the quantum dot array. The stability diagrams show the diamond-like structure characteristic of stability diagrams where regions within the diamonds correspond to stable electron configurations within the array. Each zero-bias conductance peak (Fig. S3c and f) corresponds to the addition of a single electron to the array. The observed stability diagrams are consistent with the large U approximation used since the width of these stability regions corresponds

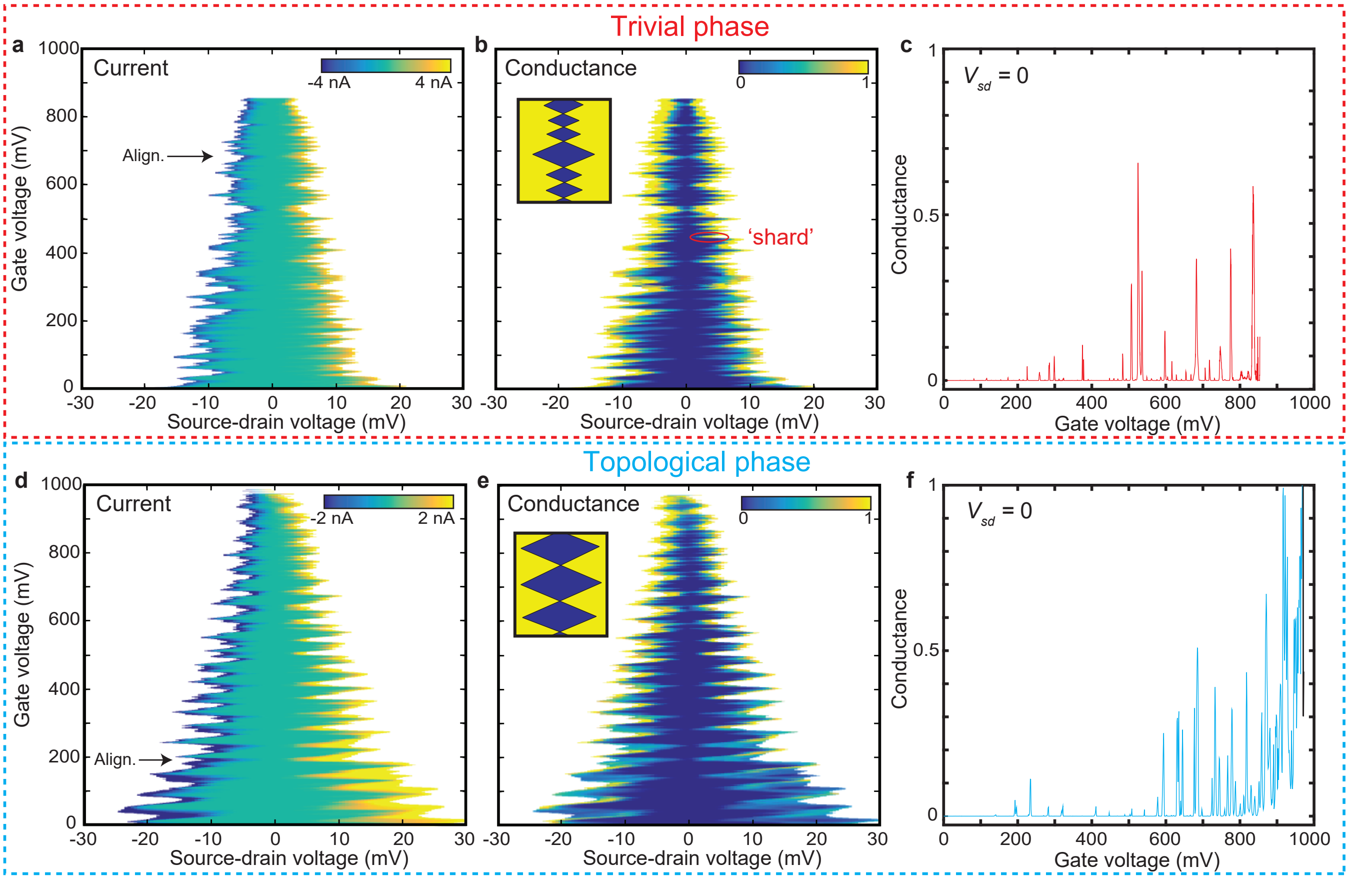

FIG. S3. **Stability diagrams for the two 10 quantum dot linear arrays (trivial and topological phases) a**, The current measured through the quantum dot array as a function of the combined gate voltages and the source-drain bias, V_{sd} for the trivial phase. The alignment procedure was performed at the indicated gate voltage. **b**, The same measurement as in **a** but for the conductance through the quantum dot array. The inset shows the 'shard' structure discussed in the text where the trivial case has many more smaller stability regions corresponding to the different electron numbers of the array. **c**, The zero-bias conductance measured over the full gate voltage range showing the many conductance peaks corresponding to the addition of single electrons to the array. **d**, The current measured through the quantum dot array as a function of the combined gate voltages and V_{sd} for the topological phase. **e**, The same measurement as in **d** but for the conductance through the quantum dot array. The inset shows the 'shard' structure discussed in the text where the topological case exhibits fewer stability regions due to the lack of current pathways away from quarter-filling. **f**, The zero-bias conductance measured over the entire gate voltage range.

to the quantum dot addition energy ($E_a \approx U_{m+1} - U_m$) and are larger than the tunnel coupling (~ 5 meV) and temperature of the array.

The main difference between the two stability diagrams is the width and structure of the Coulomb ladder. The structure of the stability diagrams is a direct consequence of the different topologies of the SSH model. The trivial phase conductance stability diagram (Fig. S3b) is narrower and does not vary in its width as a function of gate voltage as much as the topological phase (Fig. S3e). The narrower stability diagram is again a direct consequence of the many conduction pathways of the trivial phase leading to the additional 'shards' in the Coulomb ladder [2, 11]. These shards in the Coulomb ladder correspond to the additional conductance peaks away from quarter-filling ($m+5$) and three-quarter-filling ($m+15$) of the array as seen in Fig. 3b of the main text. In comparison, the topological phase has far less 'shards' since there are only 2 states per U that allows current to flow ($m+4 \rightarrow m+5$ and $m+5 \rightarrow m+6$ as shown in Fig. 3f) and as a result the stability diagram is wider with fewer peaks between the zero-bias conduction peaks.

III. THE EFFECTS OF DISORDER

In the theoretical calculations performed in the main text the data is fitted using the many-body Hamiltonian given by Equation 1 with the assumption that the energy levels of the quantum dots are tuned to account for the inter-site Coulomb interaction terms, $V_{i,j}$, such that $\epsilon_i = -\sum_j^N V_{i,j}$. In reality however, there will be various

TABLE S6. **Inter-dot distances and tunnel couplings for the trivial (device I) and topological (device II) phases.** The quantum dot size (nm^2) and inter-dot distances (nm) directly measured from the STM images and corresponding tunnel couplings (meV) determined from fitting the experimental zero-bias conductance peaks.

Trivial	d_1	d_2	d_3	d_4	d_5	d_6	d_7	d_8	d_9	d_{10}
	29.0	29.9	29.0	27.3	25.46	30.3	27.0	28.0	26.5	24.6
	$d_{1,2}$	$d_{2,3}$	$d_{3,4}$	$d_{4,5}$	$d_{5,6}$	$d_{6,7}$	$d_{7,8}$	$d_{8,9}$	$d_{9,10}$	$\langle d_v/d_w \rangle$
	7.7	10.0	7.7	10.3	7.8	10.1	7.9	10.2	7.7	0.76
	$t_{1,2}$	$t_{2,3}$	$t_{3,4}$	$t_{4,5}$	$t_{5,6}$	$t_{6,7}$	$t_{7,8}$	$t_{8,9}$	$t_{9,10}$	$\langle v/w \rangle$
	5.01	2.57	5.18	2.10	4.84	2.41	4.38	2.33	5.01	2.08
Topological	d_1	d_2	d_3	d_4	d_5	d_6	d_7	d_8	d_9	d_{10}
	22.6	21.0	21.5	23.1	25.4	20.9	21.1	26.4	26.6	21.2
	$d_{1,2}$	$d_{2,3}$	$d_{3,4}$	$d_{4,5}$	$d_{5,6}$	$d_{6,7}$	$d_{7,8}$	$d_{8,9}$	$d_{9,10}$	$\langle d_v/d_w \rangle$
	9.3	7.5	9.9	7.2	9.8	8.1	10.0	8.4	9.4	1.24
	$t_{1,2}$	$t_{2,3}$	$t_{3,4}$	$t_{4,5}$	$t_{5,6}$	$t_{6,7}$	$t_{7,8}$	$t_{8,9}$	$t_{9,10}$	$\langle v/w \rangle$
	1.77	6.21	1.19	7.35	1.27	4.16	1.07	3.29	1.66	0.265

forms of disorder present in the system such as in the absolute value of the quantum dot energy levels, in the inter-site tunnel couplings and the effect of a finite on-site Coulomb interaction term, U_i. In this section we discuss the impact each of these have in the context of both the single particle SSH model and their effects on the zero-bias conductance trace and ground state energies of the many-body SSH model for the two devices fabricated in this paper.

A. Quantum dot energy level disorder

Misalignments in tuning of the energy level terms of the quantum dots, ϵ_i can potentially lead to errors in the calculated tunnel coupling terms due to detuned conductance peaks in the experiment. Some small misalignments are inevitable and give rise to on-site disorder present in the diagonal terms of the Hamiltonian, given by the $\sum_{i=1}^{N} \epsilon_i n_i$ term in Equation 1. For the single particle SSH model on-site disorder is known to break the chiral symmetry [12] of the system resulting in a non-symmetric energy spectrum. This disorder shifts the zero energy edge states asymmetrically away from zero energy, however, they remain within the insulating band gap for disorder strengths, $\delta < w/2$, where w is the even tunnel coupling strength as shown in Fig. 1a of the main text.

For the many-body case the symmetry is also broken by this on-site disorder. Figure S4 shows the effect on the zero-bias conductance for small values of detuning, of 0.01 mV, 0.1 mV and 1 mV, on individual quantum dots 1 (Fig. S4a, e), 2 (Fig. S4b, f), 3 (Fig. S4c, g) and 5 (Fig. S4d, h) for devices in the trivial and topological phases. The detuning causes shifts in the conductance peaks such that the peak structure is no longer symmetric around zero. In the trivial phase (Fig. S4a, b, c and d) the detuning results in small shifts in the conductance peaks about their initial values. All 10 conductance peaks can still be observed as the electrons remain delocalised across the entire array, while the amount of shift for each peak varies depending on which quantum dot is detuned, while the conductance of some peaks increase as others decrease compared to that of no detuning.

In the topological case (Fig. S4e, f, g and h) only the conductance peaks corresponding to the two edge states of the array are shown as the conductance from states away from quarter-filling are not visible. For the small detunings investigated these states are still predominately delocalised within the bulk of the array and have a very low probability of existing on the edge quantum dots. As a result, tunnelling between these bulk-like states and the source/drain leads is significantly suppressed. The edge state conductance peaks are observed to be very sensitive to disorder in the energy levels of the quantum dots. Figure S4e shows that a small detuning (0.01 mV) on the edge quantum dot (Dot 1) results in a rapid decay in the strength of the conductance peaks, compared to that of no disorder, with the conductance peaks vanishing completely for larger detunings. These edge state conductance peaks are most strongly affected by detuning of the edge quantum dot 1 (and 10 not shown). This is due to the edge states being heavily involved in the current through array and where the electrons are most likely to be moving through the array. However, the absolute energy splitting between the two conductance peaks remains fairly stable due to the robustness of the topological phase to noise. Small offsets in the alignment of the middle quantum dots Fig. S4g and h can lead to asymmetry in the height of the conductance peaks, which is observed in the main text Fig. 3f. However, since multiple quantum dots can cause similar variations in the peak heights it is not possible to know

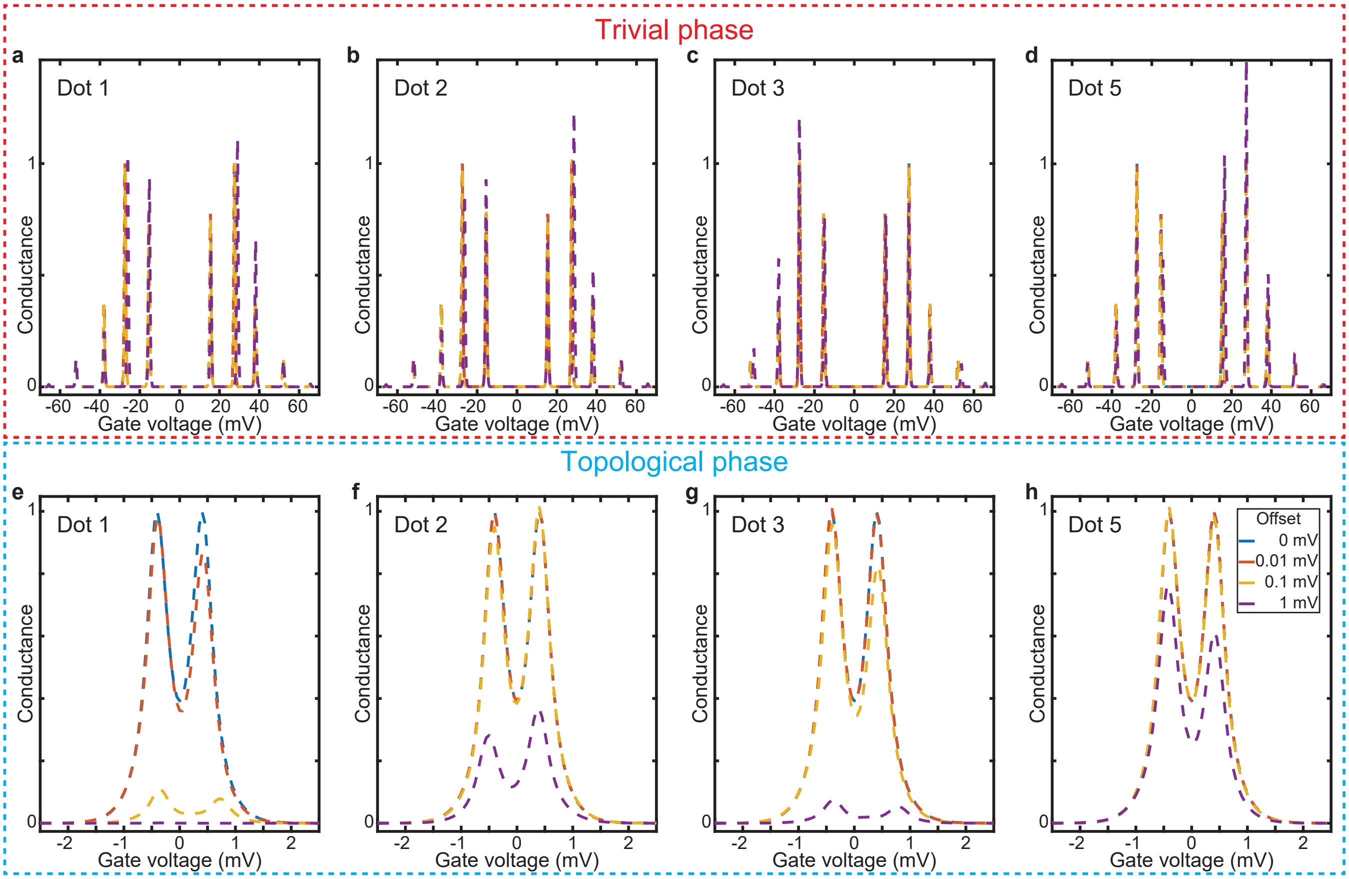

FIG. S4. **Effects of disorder in the quantum dot tuning.** Theoretical calculations of the zero bias conductance trace when an energy offset of 0.01 mV, 0.1 mV and 1 mV is applied to the first (**a**, **e**), second (**b**, **f**), third (**c**, **g**) and fifth (**d**, **h**) quantum dot along the array compared to no energy offset for the trivial phases (top panel) and topological phase (bottom) panel.

exactly which quantum dot(s) is (are) slightly misaligned.

B. Tunnel coupling disorder

To investigate the effect of the non-equal tunnel couplings throughout the array we compare the measured tunnel coupling traces to an ideal case where we have set all of the v and w couplings equal to the mean of the experimental values. In this way, the total couplings should remain the same but the experimental values will have an additional randomness to them.

In Figure S5 we show the resulting normalised theoretical conductance as a function of gate voltage for the trivial (Fig. S5a) and topological (Fig. S5b) device phases. For the trivial phase we can see that the averaged tunnel coupling and experimental parameters vary in both splittings and peak magnitudes. Similarly to the quantum dot tuning, the noise in the tunnel coupling results in small deviations from the expected ideal array where all the v and w couplings are equal. However, these variations remain relatively small with the results from the main text demonstrating exquisite control over the tunnel couplings.

For the topological phase we again see the robustness of the topological state to disorder. Indeed, some of the tunnel couplings in device II are predicted to be almost twice those of other inter-dot couplings for the v parameter due to the sensitive distance dependence (see Tab. S6). Despite this, the two conductance peaks show almost no difference in their magnitude and separation. Therefore, we can conclude that the combined alignment of the quantum dot levels and variations in the tunnel couplings are sufficient to realise the many-body SSH system.

IV. REFERENCES

[1] Watson, T. F. *et al.* Transport in asymmetrically coupled donor-based silicon triple quantum dots. *Nano Letters* **14**, 1830–1835 (2014).

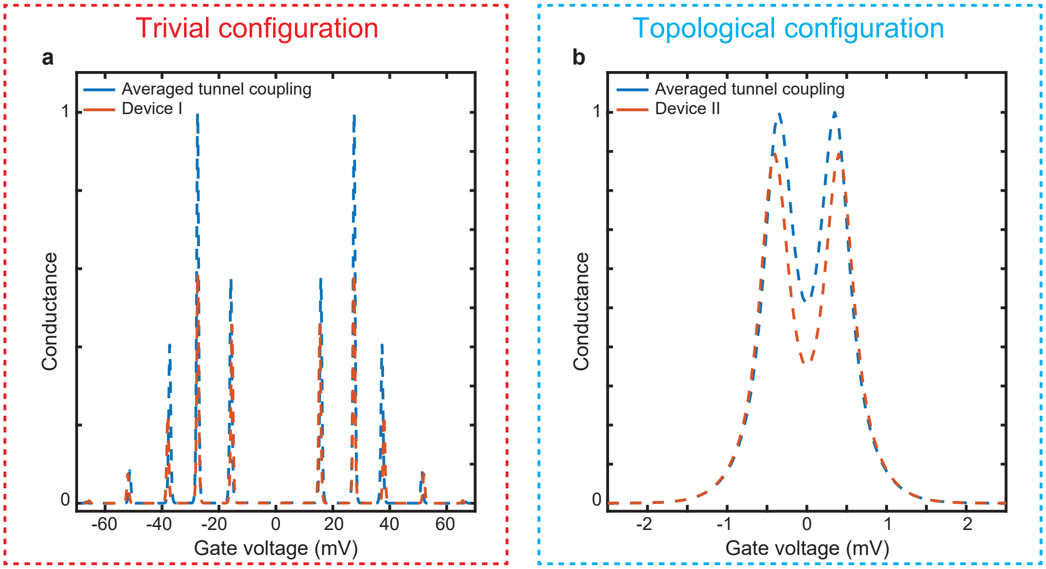

FIG. S5. **Effects of disorder in the quantum dot tunnel couplings. a**, The simulated trivial conductance as a function of gate voltage for the experimentally measured tunnel couplings and the averaged tunnel coupling where there is no variation in either v or w. The conductance traces are very similar with small differences between the magnitude and the peak-to-peak separations. **b**, The simulated topological conductance as a function of gate voltage for the experimentally measured tunnel couplings and the averaged tunnel coupling where there is no variation in either v or w. The robustness of the topological state means that even for significant variations in the individual tunnel couplings by a factor of ~ 2 obtained experimentally the conductance traces remain very similar.

[2] Weber, B. *et al.* Spin blockade and exchange in Coulomb-confined silicon double quantum dots. *Nature Nanotechnology* **9**, 430–435 (2014).
[3] van der Wiel, W. G. *et al.* Electron transport through double quantum dots. *Reviews of Modern Physics* **75**, 1–22 (2002).
[4] Weber, B., Mahapatra, S., Watson, T. F. & Simmons, M. Y. Engineering independent electrostatic control of atomic-scale (∼4 nm) silicon double quantum dots. *Nano Letters* **12**, 4001–4006 (2012).
[5] Fuechsle, M. *et al.* Spectroscopy of few-electron single-crystal silicon quantum dots. *Nature Nanotechnology* **5**, 502–505 (2010).
[6] Fuechsle, M. *et al.* A single-atom transistor. *Nature Nanotechnology* **7**, 242–246 (2012).
[7] He, Y. *et al.* A two-qubit gate between phosphorus donor electrons in silicon. *Nature* **571**, 371–375 (2019).
[8] Wang, Y., Chen, C.-Y., Klimeck, G., Simmons, M. Y. & Rahman, R. All-electrical control of donor-bound electron spin qubits in silicon. *arXiv:1703.05370* (2017).
[9] Comsol multiphysics® v. 5.6. www.comsol.com. comsol ab, Stockholm, Sweden.
[10] Byrnes, T., Kim, N. Y., Kusudo, K. & Yamamoto, Y. Quantum simulation of Fermi-Hubbard models in semiconductor quantum-dot arrays. *Phys. Rev. B* **78**, 075320 (2008).
[11] Kouwenhoven, L. P. *et al.* Electron transport in quantum dots. In *NATO-ASI Workshop on Mesoscopic Electron Transport*, 105–214 (1997).
[12] Asbóth, J. K., Oroszlány, L. & Pályi, A. *The Su-Schrieffer-Heeger (SSH) Model* (Springer, 2016).
